# Household Dysfunction Is Associated With Bullying Behavior in 10-year-old Children: Do Socioeconomic Circumstances Matter?

**DOI:** 10.1177/08862605211006352

**Published:** 2021-11-16

**Authors:** Sílvia Fraga, Sara Soares, Flávia Soares Peres, Henrique Barros

**Affiliations:** 1 EPIUnit - Instituto de Saúde Pública da Universidade do Porto, Portugal

**Keywords:** bullying, household dysfunction, school-aged children, socioeconomic circumstances, adverse childhood experiences

## Abstract

This study measured the prevalence of bullying behavior in 10-year-old children and investigated the effect of the socioeconomic context on the impact of household dysfunction on bullying. We studied 5,338 members of the Portuguese Generation XXI birth cohort. Information on involvement in bullying, socioeconomic characteristics, and household dysfunction was collected by trained interviewers using structured questionnaires. Being a victim of bullying was reported by 14.4% of participants, being a bully by 1.4%, and being a bully-victim by 3.9%. Being a victim or both bully-victim, simultaneously, was more frequent among children from medium-high income families. Also, children from low-income families who reported household substance abuse, witnessed parents’ intimate partner violence, and were victims of physical violence, were more frequently victims of bullying; and those who experienced family violence were more frequently involved as bully-victims. Among children from medium-high income families, all these household adversity experiences significantly increased the odds of being victim, bully, or bully-victim. Thus, although children from medium-high income families are less likely to experience adversity at home, when it happens, there is a greater effect on their behavior, suggesting that better socioeconomic circumstances do not seem to act as a protective factor.

## Introduction

Bullying behavior is a global phenomenon and highly prevalent in early adolescence (UNICEF, 2018), with devastating consequences and health implications. Several authors have provided definitions of bullying, with Farrington defining it as a “repeated oppression, psychological or physical, of a less powerful person by a more powerful one” (Farrington, 1993) and Coloroso defining bullying as “a conscious, deliberate hostile activity intended to terrorize and harm others through the threat of further aggression” (Coloroso, 2008). The most common and widely accepted definition of bullying was described by Olweus and states that “a person is bullied when he or she is exposed, repeatedly and over time, to negative actions on the part of one or more other persons” (Olweus, 1993). However, in all these definitions, authors agree that bullying is a form of aggression with serious long- and short-term implications on health and wellbeing of those who are involved.

A bulk of the literature have linked bullying victimization with a wide range of adverse health outcomes, mainly related to anxiety, depression, self-injury, and suicidal ideation (Lereya et al., 2015; Moore et al., 2017), and with engagement in unhealthy risk behaviors (Hertz et al., 2015; Moore et al., 2017; Waasdorp et al., 2018). Also, bullying others is predictive of poor academic achievement (Nansel et al., 2001, 2004), antisocial personality and other psychiatric disorders, substance abuse, and suicidal ideation in adulthood (Klomek et al., 2009; Sourander et al., 2007, 2009, 2011; Ttofi et al., 2011).

### Bullying Forms

Bullying can take the form of verbal abuse, physical violence, or social rejection. Some authors also consider that these forms of bullying entail direct (verbal and physical) or indirect (relational) bullying (Björkqvist et al., 1992).

The direct or more overt aggression includes observable confrontations which involve physical and verbal attacks; while indirect bullying includes attacks that are carried out in a more covert and secretive manner, using spreading rumors, excluding people from groups, or persuading or daring a peer to harm another child or adolescent.

Gender seems to play an important role in bullying involvement (Steinfeldt et al., 2012). There is evidence showing that the rates of aggressive behavior and bullying behavior are higher among boys than girls (World Health Organization, 2017). However, some researchers argue that there is not a gender difference between boys and girls, but instead the two groups use different forms of aggression. Evidence suggests that boys are more likely to be involved as victims or as bullies, especially in its physical expression (Craig & Harel-Fisch, 2004; Silva-Rocha et al., 2020), while girls are more likely to engage in situations of indirect bullying, such as teasing or gossiping (Committee on the Biological and Psychosocial Effects of Peer Victimization, 2016; Finkelhor et al., 2015; Silva-Rocha et al., 2020). This difference in behavior might be attributed to biological reasons, or to gender differences in the socialization process, where boys are encouraged to be aggressive and competitive, but girls are expected to be nurturant and expressive. However, the consequences of bullying for the well-being and in health outcomes are not expected to be different among girls and boys.

### Household Environment and Bullying Behavior

The social environment influences the nature and quality of social relationships which in turn affect child development and future achievements. Literature has shown that a warm, authoritative, and sensitive parenting style contributes to children’s positive social behavior and supportive peer relationship (Helsen et al., 2000). Also, students with higher “self-confidence” and “avoidance from bullying” scores, are more likely to report lower levels of family dysfunctions (Eşkisu, 2014).

On the other hand, a harsh family environment, with a low parental capacity to care for their kids, may affect children’s social skills and decrease their ability to cope with stress and difficult times (Álvarez-García et al., 2015; Hong & Espelage, 2012; Mazur et al., 2017; Yeager et al., 2015). Authoritarian parenting styles, such as use of physical discipline, and hostile and rejecting parenting influence engagement in bullying behavior through the development of poor self-image and emotional dysregulation (Christie-Mizell, 2003; Espelage & Swearer, 2003; Espelage et al., 2003; Shields & Cicchetti, 2001). Also, the exposure to household conflicts poses a significant threat to children’s ability to process and regulate emotions, and it may result in undercontrolled or overcontrolled emotional reactions contributing to both internalizing and externalizing behaviors (Zarling et al., 2013).

A dysfunctional family environment is associated with the onset of episodes of anxiety, aggressiveness, and impulsivity in the children, impairing their development tactics to solve a conflict with their peers (Kolk, 2017), and putting them at greater risk of displaying violent, aggressive, and bullying behaviors outside the home environment (Hong & Espelage, 2012). Children who bully others are more likely to come from family environments characterized by less cohesion, expressiveness, organization, control, and social orientation (Stevens et al., 2002). In addition, although parents’ aggressive punishment did not influence the child’s aggressive behavior directly, child’s aggressive behavior depends on the parents and child relationship. Parents of aggressive boys expressed higher approval of aggression in social life (Frączek & Kirwil, 1992) and children who are raised by dominant parents or with an over-control in their behavior tend to harass their classmates at school (Manning et al., 1978). At the same time, permissive parents legitimize combative activities and fail to provide opportunities for the child to control his or her aggressive urges (Schaffer, 1994).

Accordingly, growing up in a dysfunctional environment adversely impacts the children’s behavior. However, the specific nature of this link remains unclear. There are studies that find links to internalizing behaviors while other show links to externalizing behaviors (Kitzmann et al., 2003). Although we will not explore which mechanisms would explain these links, it would be relevant, therefore, to understand how this household dysfunction impact bullying behavior in children, in terms of frequency of aggression and victimization.

### Socioeconomic Circumstances, Household Dysfunction, and Bullying

Studies suggested an inconsistent relationship between low socioeconomic circumstances and a higher likelihood of being victims and being involved as bully-victims simultaneously. While some authors state that family socioeconomic circumstances influence bullying involvement, and children from low socioeconomic backgrounds and living with a single parent are more likely to be involved in bullying behaviors (Nordhagen et al., 2005), a systematic review showed that the association of socioeconomic circumstances with bullying was weak, providing little guidance for targeted interventions (Tippett & Wolke, 2014).

However, a clear relationship between socioeconomic circumstances in childhood and neglect or maltreatment was reported in a systematic review, suggesting that low childhood socioeconomic circumstances is a determinant of such adversity (Walsh et al., 2019), and the longitudinal nature of many studies support a causal association. So, low socioeconomic circumstances seem to increase a child vulnerability to abuse and neglect at home (Ondersma, 2002). This can contribute to the development of low interpersonal skills, which in turn might increase the likelihood of involvement in bullying behaviors at school (Bowes et al., 2009; Glew et al., 2005; Jansen et al., 2011, 2012; Tippett & Wolke, 2014). It would be of relevance to investigate the impact of household dysfunction on bullying behavior considering the socioeconomic context in which children are living.

### This Study

We hypothesize that the experience of household dysfunction by children from an advantaged socioeconomic group would less likely lead to engaging in peer violence. Studies have encouraged monitoring the different environments in which children are exposed, to provide a better understanding of the mechanisms and determinants of bullying involvement at early ages, and consequently to provide evidence to intervene and prevent its adverse consequences (Garmy et al., 2018; Henderson et al., 2018). Thus, we measured the prevalence of bullying involvement in 10-year-old children and examined how household dysfunction was associated with bullying behavior considering different family socioeconomic contexts.

## Methods

### Study Design and Participants

This study was conducted in the Generation XXI cohort, a prospective Portuguese population-based birth cohort. Briefly, the cohort was assembled between 2005 and 2006 and recruited a total of 8,647 newborn from the five public maternity units of Porto Metropolitan Area providing obstetrical and neonatal care covering, at the time. All maternities were level III units, with differentiated perinatal support, and in 2004, were responsible for 91.6% of the deliveries in the whole catchment population, with the remaining occurring in private hospitals/clinics (Alves et al., 2012; Larsen et al., 2013). The entire cohort was invited to participate in the following study waves, at children’s ages of 4, 7, and 10 years old (in 2009-2011, 2012-2014, and 2016-2017, respectively). Participants do not receive financial incentives for participation in the cohort evaluations, but the research team sends a report from the doctor comprising blood collection analysis and results from physical examination. In all study waves, information on demographic and socioeconomic characteristics, obstetric history, history of disease, and health-related behaviors were collected by trained interviewers using structured questionnaires. The present is a cross-sectional study that analyses data from 5,338 participants of the fourth study wave of Generation XXI, with complete information on bullying involvement collected at the age of 10 years.

Generation XXI was approved by the Portuguese Data Protection Authority and the Ethics Committee of the University of Porto Medical School/S. João Hospital Centre approved the study protocol (CES-01/2017). Informed consent was obtained for all participants, signed by their legal guardians at every study wave (Alves et al., 2012).

### Bullying Behavior

Bullying behavior was self-reported and assessed through the Bully Scale Survey developed by the Centers for Disease Control and Prevention (CDC) (Hamburger et al., 2011). This scale collects information on the experience of bullying as a victim (11 items) and as a bully (11 items), as described in Supplementary Table 1. For each item, the child had to indicate the frequency of involvement, through five options: “never,” “rarely,” “sometimes,” “often,” and “always.” As bullying is a repeated behavior, we considered that it had happened when the child reported that at least one act occurs with the frequency “often” or “always.” Bullying involvement was categorized as “victim,” when the child answered “often” or “always” in the victimization scale, but answered “never,” “rarely,” or “sometimes” in the aggression scale; the child was classified as a “bully” when answered “often” or “always” in the aggression scale, but answered “never,” “rarely,” or “sometimes” in the victimization scale; or as “bully-victim” when was involved both as a victim and as bully simultaneously.

### Household Dysfunction

Household dysfunction was assessed through a list of stressful events that were reported by the child at the age of 10 years and to which the child was asked to answer if it has ever occurred, with “yes” or “no” answers. Household dysfunction comprised: “household substance abuse” if the child reported living with a household member with problems with alcohol and/or drug abuse; “household criminality,” if the child reported the imprisonment of a relative, “witness parents intimate partner violence (IPV).” At the same wave, maternal history of victimization was reported by the mothers and defined as lifetime experience of emotional or physical abuse occurring during adulthood. Exposure to physical violence at the age of 10 years was assessed using a single item question: *“Did someone in your house hit, kick, or punch you?”*

### Socioeconomic Circumstances

The household income included salaries and other sources of income, such as financial assistance, rent, monetary allowances, and alimony for the whole household. A low disposable household income was defined as €1,000 per month or less; intermediate if between €1,001 and €2,500, and high if more than €2,500. For analysis purposes, household income was dichotomized into “less or equal to 1,000 euros” corresponding to low income, and “more than 1,000 euros” per month corresponding to medium-high income.

### Covariates

Data on family structure, parental education, and parental history of employment were reported by the parents at wave 4 when the child was 10 years old. Children were divided into the following two categories: “living with both parents” and “living with one parent/none of the parents.” Parents educational level was measured as the number of years of formal schooling completed and classified according to the International Standard Classification of Education 2011 classes (Unesco Institute for Statistics, 2012). The low educational level corresponded to 9 years or less of formal schooling; intermediate education to 12 years of formal education; and high education to more than 12 years of formal education. For analysis, parents’ educational level was dichotomized into low if “equal or lower than 9 years” and medium-high if “more than 9 years” of formal schooling. History of parental unemployment status was coded as “yes” if at least one of the parents reported having been unemployed at least for 12 months since 2009 and “no” for all other cases.

### Statistical Analysis

All statistical analyses were performed using the software STATA version 15.1 (Stata Corp. 2017. Stata Statistical Software: Release 15. College Station, TX: StataCorp LLC). Pearson’s chi-squared test was used to compare proportions. To identify the association between household dysfunction and being bullied, logistic regression analyses were run. We calculated odds ratios, with 95% confidence intervals [*OR*, 95% CI]), sex-adjusted odds ratios (AOR), and 95% CI for the studied association. The primary outcome was involvement in bullying, as a victim, bully, or bully-victim. Children’s sex was considered as a confounder in the association between household dysfunction and type of involvement in bullying to account for some gender differences in the bullying tactics. Analyses were stratified by household income, as we previously found a significant interaction of household income *household dysfunction*bullying.

## Results

Overall, 19.7% of children reported to have been involved in bullying at the age of 10 years; involvement as a victim was reported by 14.4% of the Generation XXI participants, involvement as a bully by 1.4%, and involvement as bully-victim by 3.9%. Boys were more frequently involved in bullying behaviors than girls (16.6% versus 12.0% as victims; 2.0% versus .7% as bullies; and 5.5% versus 2.3% as bully-victims).

Table 1 shows that children involved as a victim or as a bully-victim presented more frequently parents with lower levels of formal education and history of unemployment, belonged to families with a low household income or lived in a one-parent/none of the parents’ family structure. Similarly, the report of household member with problems with alcohol and/or drug abuse, criminality, witnessing parents IPV, and maternal history of victimization were more frequent among children involved in bullying behaviors. Also, children exposed to physical violence at home were more frequently involved in bullying behaviors when compared to those who were not exposed to physical violence (Table 1).

**Table 1.** Sociodemographic Characteristics, Household Dysfunction, and Exposure of the Child to Physical Violence by Type of Involvement in Bullying (Not-involved, Victim, Bully, and Bully-Victim).

	Bullying Involvement, *n* (%)				
	Not-involved	Victim	Bully	Bully-Victim	
	80.3%	14.4%	1.4%	3.9%	*P*-value
Child sex					
Girl	2,223 (85.0)	315 (12.0)	18 (.7)	61 (2.3)	.001
Boy	2,066 (75.9)	451 (16.6)	54 (2.0)	150 (5.5)	
Child age, mean (±*SD*)	10.1 (.3)	10.1 (.3)	10.1 (.3)	10.2 (.3)	
Parental education					
Low (≤9th grade)	956 (78.0)	193 (15.8)	16 (1.3)	60 (4.9)	<.001
Medium-high (>9th grade)	2,442 (83.4)	361 (12.3)	38 (1.3)	88 (3.0)	
Parental unemployment					
No	2,385 (83.8)	339 (11.9)	39 (1.4)	82 (2.9)	<.001
Yes	1,003 (77.3)	215 (16.5)	15 (1.2)	65 (5.0)	
Household Income					
Low (≤€1,000/month)	1,057 (76.1)	245 (17.7)	18 (1.3)	68 (4.9)	<.001
Medium-high (>€1,000/month)	308 (82.1)	489 (13.0)	49 (1.3)	134 (3.6)	
Family structure					
Both parents	3,400 (81.9)	552 (13.3)	54 (1.3)	146 (3.5)	<.001
One parent/other structure	884 (75.0)	211 (17.9)	18 (1.6)	65 (5.5)	
Household substance abuse					
No	4,240 (80.8)	734 (14.0)	71 (1.4)	202 (3.8)	<.001
Yes	48 (53.9)	31 (34.9)	1 (1.1)	9 (10.1)	
Household criminality					
No	4,159 (80.8)	726 (14.1)	66 (1.3)	195 (3.8)	<.001
Yes	129 (67.6)	40 (20.9)	6 (3.1)	16 (8.4)	
Witnessing parents IPV					
No	2,553 (86.3)	303 (10.2)	29 (1.0)	74 (2.5)	<.001
Yes	1,724 (73.0)	460 (19.5)	43 (1.8)	136 (5.7)	
Maternal history of victimization					
No	2,787 (82.2)	451 (13.3)	40 (1.2)	113 (3.3)	<.001
Yes	779 (74.0)	186 (17.6)	21 (2.0)	68 (6.4)	
Child exposure to physical violence					
Never	1,054 (90.9)	87 (7.5)	4 (.4)	14 (1.2)	<.001
Yes	3,235 (77.4)	678 (16.2)	68 (1.6)	197 (4.7)	

Table 2 shows that children from low-income families, who report household substance abuse and witness parents IPV, are exposed to physical violence at home and whose mothers report “history of victimization” were more frequently involved in bullying behaviors. The same results were found among children from medium-high income families. However, the family structure and history of household criminality were also related to bullying involvement in children from medium-high income families (Table 2).

**Table 2.** Sociodemographic Characteristics, Household Dysfunction, and Exposure of the Child to Physical Violence by Type of Involvement in Bullying, According to Household Income.

Household Income										
	Low (≤€1,000)*n* (%)					Medium-High (>€1,000)*n* (%)				
	Involvement in Bullying					Involvement in Bullying				
	Not-involved	Victim	Bully	Bully-Victim	*P*-value	Not-involved	Victim	Bully	Bully-Victim	*P*-value
Family structure										
Both parents	641 (77.5)	136 (16.4)	8 (1.0)	42 (5.1)	.262	2,671 (83.1)	403 (12.5)	41 (1.3)	100 (3.1)	<.001
One parent/other structure	416 (74.2)	109 (19.4)	10 (1.8)	26 (4.6)		413 (76.3)	86 (15.9)	8 (1.5)	34 (6.3)	
Household substance abuse										
No	1,035 (76.8)	232 (17.2)	17 (1.3)	64 (4.7)	.031	3,061 (82.5)	471 (12.7)	49 (1.3)	129 (3.5)	<.001
Yes	22 (56.4)	12 (30.8)	1 (2.6)	4 (10.2)		23 (50.0)	18 (39.1)	0 (0)	5 (10.9)	
Household criminality										
No	977 (76.7)	222 (17.5)	15 (1.2)	59 (4.6)	.176	3,041 (82.4)	476 (12.9)	46 (1.2)	128 (3.5)	.001
Yes	80 (69.6)	23 (20.0)	3 (2.6)	9 (7.8)		42 (65.6)	13 (20.3)	3 (4.7)	6 (9.4)	
Witness parents IPV										
No	604 (83.1)	89 (12.2)	10 (1.4)	24 (3.3)	<.001	1,858 (87.6)	196 (9.2)	18 (.9)	48 (2.3)	<.001
Yes	446 (68.4)	154 (23.6)	8 (1.2)	44 (6.8)		1,222 (75.0)	292 (17.9)	31 (1.9)	85 (5.2)	
Maternal history of victimization										
No	605 (78.5)	131 (17.0)	7 (.9)	28 (3.6)	<.001	2,084 (83.5)	302 (12.1)	30 (1.2)	79 (3.2)	<.001
Yes	274 (71.0)	70 (18.1)	10 (2.6)	32 (8.3)		483 (75.8)	109 (17.1)	10 (1.6)	35 (5.5)	
Child exposure to physical violence										
No	282 (87.0)	33 (10.2)	0 (0)	9 (2.8)	<.001	729 (92.9)	48 (6.1)	3 (.4)	5 (.6)	<.001
Yes	775 (72.8)	212 (19.9)	18 (1.7)	59 (5.5)		2,355 (79.3)	440 (14.8)	46 (1.5)	129 (4.3)	

Table 3 shows that children from low-income families who reported household substance abuse (*OR* = 2.23, 95% CI: 1.08–4.59), witnessed parents IPV (AOR = 2.30, 95% CI: 1.72–3.07), and exposed to physical violence (AOR = 2.20, 95% CI: 1.49–3.27) were more likely to be victims of bullying. Family violence as parents IPV, maternal victimization, and child exposure to physical violence was associated with involvement in bully-victim behaviors. The previous history of maternal violence (AOR = 3.18, 95% CI: 1.20-8.46) was the only adverse experience that was associated with involvement as a bully. However, among children from medium-high income families, household dysfunction experiences were statistically significantly associated with bullying behavior. Living in a one-parent/none of the parents family structure, history of household criminality, witnessing parents IPV, history of maternal violence, and child exposure to physical violence was associated with being a victim or a bully-victim. Household criminality (*OR* = 4.97, 95% CI: 1.47-16.82), witnessing parents IPV (*OR* = 2.38, 95% CI: 1.32-4.28), and child exposure to physical violence (*OR* = 4.29, 95% CI: 1.33-13.85) were associated with being a bully in children from medium-high income families (Table 3).

**Table 3.** Sex-adjusted Odds Ratios (*OR*) and 95% Confidence Intervals (CI) for the Association of Household Dysfunction With the Type of Involvement in Bullying, According to Low and Medium–High Household Income.

Household Income						
Low Income				Medium-High Income		
	Victim	Bully	Bully-Victim	Victim	Bully	Bully-Victim
Household substance abuse	2.23 (1.08**-**4.59)	2.47 (.31**-**19.53)	2.64 (.88**-**7.94)	4.81 (2.57**-**9.02)	**–**	4.59 (1.69**-**12.36)
Household criminality	1.29 (.79**-**2.11)	2.52 (.71**-**8.90)	1.92 (.91**-**4.02)	2.02 (1.07**-**3.80)	4.97 (1.47**-**16.82)	3.55 (1.47**-**8.61)
Witness parents IPV	2.30 (1.72**-**3.07)	1.05 (.41**-**2.70)	2.42 (1.45**-**4.05)	2.18 (1.80**-**2.66)	2.38 (1.32**-**4.28)	2.47 (1.72**-**3.55)
Maternal history of victimization	1.19 (.86**-**1.65)	3.18 (1.20**-**8.46)	2.55 (1.50**-**4.32)	1.59 (1.25**-**2.02)	1.52 (.74**-**3.14)	2.01 (1.33**-**3.03)
Child exposure to physical violence^*^	2.20 (1.49**-**3.27)	**–**	2.21 (1.08**-**4.54)	2.73 (2.00**-**3.72)	4.29 (1.33**-**13.85)	7.31 (2.98**-**17.95)

*Note*. ^*^Exposed at age of 7 and 10 years.

## Discussion

This study showed that although children from medium-high income families were less likely to experience adversity in their home and engage in violent behaviors, when stressful household events occur it substantially impacts the odds of being involved in bullying behaviors. Though we have to be careful when establishing comparisons between these groups of participants since their basal risk may be different, our results seem to support that the negative influence of household adversity in children’s behavior is not only limited to children in low socioeconomic families. As previously reported, being exposed or witnessing other forms of victimization at home might increase their susceptibility for being involved in bullying (Lereya et al., 2015), as children might see it as an acceptable way to manage interpersonal conflicts. When children from higher socioeconomic groups suffer an adverse experience in their home, the effect on their behavior is greater, suggesting that a more favorable socioeconomic environment may not act as a protective factor against the influence of household dysfunction. A similar finding was reported in a previous study (Halfon et al., 2017), in which authors showed that children in the highest income bracket who suffer from adverse childhood experiences were not protected against the effect of these adverse experiences on their health. Thus, a higher income does not always protect a family from the impact of a stressful event. On the other hand, if a child lives in a disadvantaged socioeconomic environment and is also exposed to material deprivation or financial strain, with the daily stress related to socioeconomic deprivation and its consequences, the impact of another traumatic psychosocial event may not have much meaning; but if a child belongs to a high socioeconomic household and experiences a traumatic event, this child will have increased levels of stress that may influence child’s reactions and behaviors, which may explain engagement in bullying. Moreover, we must acknowledge the intergenerational transmission of disadvantage that states that adverse family-related circumstances are associated with increased odds of experiencing disadvantaged social, economic, and health-related trajectories across adulthood. Thus, our results are in line with the theories of cumulative disadvantage, reflecting that an adverse situation in childhood may be a marker for accumulated and, to some extent, persistent problems later in life (Almquist & Brännström, 2018).

Previous studies showed that victims of bullying and bully-victims were more likely to come from low socioeconomic households (Alikasifoglu et al., 2007; Bowes et al., 2009; Jansen et al., 2011; Jansen et al., 2012; Tippett & Wolke, 2014). Our results also showed that household income was associated with bullying behavior, with those children from low socioeconomic conditions reporting more frequently being a victim or a bully-victim. Besides, we observed an increased likelihood of involvement as a bully in children from medium-high income when the history of household criminality, parents IPV, and exposure to physical violence is reported. These findings suggest that exposure to household dysfunction might impact children’s emotional and behavioral development, and as previously reported, with later increased risk of mental health consequences (Lereya et al., 2015) and disease conditions (Felitti et al., 1998).

In the study of Lereya et al., it was observed that children who were bullied were more likely to have mental health problems later in life, while children who were both maltreated and bullied were also at increased risk for mental health problems, but with lower risk than those of being bullied alone. Thus, being bullied had worse long-term adverse effects on young adults’ mental health than being maltreated by adults (Lereya et al., 2015). Taking these results into account, investments in the identification of children at risk of becoming a bully or victim could allow the intervention at younger ages and timely prevention of bullying and victimization, and consequently of its potential detrimental health outcomes. Identification is enhanced by knowledge on determinants and predictors of bullying behavior. Also, as involvement in bullying seems to be socially patterned, family, school, and neighborhood socioeconomic circumstances might also help to identify an increased risk of involvement and predict bullying behavior as these factors are likely to influence children’s behavior, and pose as a window of opportunity to intervene. Nevertheless, some controversy remains on the effectiveness of an intervention in school environments to prevent bullying. Even though several research teams have studied the effectiveness of various bullying prevention programs, a meta-analysis by Ttofi and Farrington is recognized as one of the most comprehensive and rigorous to date. They conclude that whole-school programs are effective in reducing bullying and victimization but also that there are great variations in the effects of different programs (Ttofi & Farrington, 2009).

Our study found that one in five children have been involved in repeated behaviors of bullying and that boys were more frequently involved in bullying than girls. This finding might mean that boys are more willing to report their bullying behaviors, or they are more willing to use bullying as a dominant strategy. Nevertheless, our results showed a higher prevalence of pure victims among boys when compared to girls. This is contrary to what most previous studies have shown, in which girls were more likely to report higher levels of victimization. Also, the prevalence of being a bully or being bully-victim is lower in our study. As they grow, children who were victims of bullying would be more prone to get involved in bullying others and, therefore, to be both victims and bullies simultaneously. Although our study is focused on a particular age, it is expected that the prevalence of pure victim’s decreases, and the prevalence of bully-victims and pure bullies increases with age (Craig et al., 2009).

One of the main strengths of this study is the use of a large study, such as Generation XXI. The use of several detailed questions about exposures to a household with dysfunctions, as well as data on bullying involvement, through a detailed scale, is likely to have given a higher catchment rate for those exposures than single screening questions. The information on adverse experiences was self-reported, so the children completed the questionnaire by themselves. It allowed us to assess self-reported involvement instead of the involvement reported by parents or professors as in other studies (Bowes et al., 2009; Foster & Brooks-Gunn, 2013). Also, the interviewer should only intervene when the child asked for help; therefore, the presence of the interviewer is not expected to influence children’s answers. However, self-report answers can add ambiguity due to undisclosed or socially desirable answers, especially expected when dealing with these private issues. Thus, it could contribute to underestimate the prevalence of bullying. Furthermore, we emphasize that while other studies are limited to investigate specific indicators such socioeconomic (Magklara et al., 2012) or other stressful or violent events (Bowes et al., 2009), our results exhibit a broader approach, once it takes into account the socioeconomic environment and the exposure to household dysfunctions simultaneously, which may be more useful to predict and prevent bullying involvement.

Our sample includes participants of Generation XXI, that were not selected by gender, ethnicity, or religion. Moreover, Generation XXI participants are almost exclusively Caucasian, and there is no ethnic variability to account for. In our cohort, less than 5% of mothers were born in another country. The lack of diversity at the recruitment time is explained by the small number of foreign citizens (329,898) who were living in Portugal in 2006, and by the fact that most of them were concentrated in the metropolitan area of Lisboa (Instituto Nacional de Estatística, 2007). As it is not expected that exposure to bullying varies for different ethnic or religious groups, these concepts are not to consider in the observed results, and the associations we found are mainly due to exposure to household adversity and socioeconomic circumstances differences.

However, some limitations must be acknowledged. Generation XXI aims at attaining novel and useful knowledge for understanding the Portuguese reality, by using data from children born in the metropolitan area of Porto, and by thus predominantly urban. However, it is not expected that the main results would be different if including children from rural areas. Also, within the cohort, we assessed bullying involvement and household adversity at the age of 10 years, and we used family socioeconomic circumstances reported at the same time, precluding any comparison or causality estimation due to cross-sectional nature of the study. Nevertheless, variables used as exposures, such as household dysfunction are considered as risk factors and cannot be consequences of bullying involvement. Even though exposure of the child to violence can be framed as either risk factor or consequence of bullying, we believe that family environment seems to play a substantial role in the likelihood of a child to become involved in bullying (Álvarez-García et al., 2015; Hong & Espelage, 2012; Mazur et al., 2017; Yeager et al., 2015) and thus explaining the association found. Therefore, it would be particularly informative for future studies to examine these exposures and bullying involvement by using longitudinal data. Additionally, although there is a potential bias through underestimation when asking for family income (Moore et al., 2000), the existence of bias would lead to an underestimation of the associations, therefore, not affecting our results. Moreover, we used data aggregated into classes instead of the format used for data collection, to easier comprehension and statistical efficiency. This way we can compare participants from low with medium-family incomes and also, identify different types of involvement in bullying, and classifying them according to the frequency of the involvement. Even though it is quite common to combine subjects with some score above a threshold into categories, as we did to the income and bullying variables, taking advantage of increasing the prevalence of participants in the different categories and improving the ease of comprehension and the statistical efficiency of the analysis, they may come at the price of misclassification of participants. We believe that this option does not affect our results, since we collapsed only adjacent categories with similar frequencies (in the case of bullying) and similar proportions (in the case of income). Thus, the impact of misclassification should be residual in the analyses. Also, as a common occurrence in prospective birth cohorts, there has been attrition over time, leading to a reduction in the sample size and a more socioeconomically advantaged group of participants throughout childhood and cohort evaluations. Nevertheless, we believe that the inclusion of the more-disadvantaged group would have widened the differences observed. The number of missing’s in the specific questions regarding bullying is insignificant and by thus, does not affect our findings.

We must also acknowledge that some dimensions might be important to take into account in future studies addressing this association. Perceived social support, meaning the knowledge and feeling that a person is cared for (Davidson & Demaray, 2007), ensure positive effects and may act as a “buffering effect” that can mitigate the negative impact of stress and exposure to a problematic situation (Holt & Espelage, 2007). Thus, social support can improve the coping ability and reduce the harmful consequences of bullying (Eşkisu, 2014), conducting toward a process of building resilience. Resilient individuals are those who manifest positive outcomes over time despite facing significant adversities (Luthar et al., 2000). Peer relationships may also play a role in promoting resilience to bullying. For example, bullied adolescents who report high levels of support from peers are more likely to maintain appropriate academic achievement for their age group compared to those with low peer support (Rothon et al., 2011; Wang et al., 2011).

## Implication of Findings

Growing up and living in a dysfunctional context may contribute not only for learning negative relationship patterns but also to compromise the child’s healthy development by making them more vulnerable (Felitti et al., 1998; Lereya et al., 2015). Although children from medium-high income families are less likely to experience adversity in the home, when it happens, the effect on their behavior is greater, which suggests that better socioeconomic circumstances do not seem to act as a protective factor. However, we should focus on the high prevalence of bullying among 10-year-old children, those living and growing up in poverty, and on how psychosocial events may be managed by children. Bullying prevention and intervention strategies should target all the children, independently of their socioeconomic origin. Besides, children who have experienced a life stressful event should have the opportunity to receive psychological support.

Even though, in terms of implications, creating and sustaining safe, stable, nurturing relationships and environments for all children and families, independently of their socioeconomic background, may contribute not only to prevent adversity but also to provide children with the ability to cope with adversity. Furthermore, an investment in strategies for preventing and stopping bullying that entail a commitment of families, schools, and community would contribute to raising awareness of this phenomenon, and children should be more prepared to identify it as a problem and to manage to cope with their conflicts without engaging in violent behaviors. Particularly, reduction of bullying must aim multiple levels of the social-ecology and by extension, by contributions from individuals across a range of disciplines. At the family level, the implementation of programs that include a parent- or family-focused components, such as parent-child communication about violence and bullying behaviors; at school level promoting popular and political debates, rendering educational research on bullying among children and adolescents; and at the community level, media and mass communications, motivated by popular debate criticizing and condemning violence and bullying behaviors. Finally, special attention should be given to children from poor families who were likely to have an inferior quality of life.

To sum up, our findings raise awareness for a greater investment in bullying prevention involving not only schools, families, and children but also the entire community. Schools or community-based organizations should be able to provide a mechanism of support for those children who experienced life stressful events at home. Although our results show a high impact of household adversity on bullying involvement among those from favorable socioeconomic environments, it does not mean that children from lower socioeconomic groups do not need to receive psychological support. They also suffer when a stressful event occurs, but poverty has a higher impact on their lives for much longer. If children are already in a trajectory of adversity, caused by experiencing adverse circumstances in the family of origin that will relate to the child social, economic, and health-related disadvantages across the lifecourse, another negative event in early life will not have a multiplicative effect but an additive contribution to the suffering level. Finally, school environments should contribute to reducing inequalities and therefore children from poorer social backgrounds should not be left behind.

## Author Biographies

**Sílvia Fraga**, PhD in Public Health, is a Social Epidemiologist at EPIUnit-Instituto de Saúde Pública da Universidade do Porto, Portugal. Her research focuses on social determinants of health, in particular on the contribution of social adverse experiences such as interpersonal violence to health inequalities.

**Sara Soares**, PhD in Public Health, is conducting her research at EPIUnit-Instituto de Saúde Pública da Universidade do Porto. Her research focuses on the biological consequences of social adversity in childhood.

**Flávia Soares Peres**, MSc in Public Health with specialization in Epidemiology, is a Research Fellow at the Social Epidemiology Group at EPIUnit-Instituto de Saúde Pública da Universidade do Porto.

**Henrique Barros**, MD, PhD, is a full professor at University of Porto, and researcher at EPIUnit-Instituto de Saúde Pública da Universidade do Porto, Portugal. His research interests focus on perinatal epidemiology and child health.

## References

[bibr1-08862605211006352] Alikasifoglu M., Erginoz E., Ercan O., Uysal O., & Albayrak-Kaymak D. (2007). Bullying behaviours and psychosocial health: Results from a cross-sectional survey among high school students in Istanbul, Turkey. *European Journal of Pediatrics*, 166(12), 1253–1260. https://doi.org/10.1007/s00431-006-0411-x 17273831 10.1007/s00431-006-0411-x

[bibr2-08862605211006352] Almquist Y. B., & Brännström L. (2018). Childhood adversity and trajectories of disadvantage through adulthood: Findings from the stockholm birth cohort study. *Social Indicators Research*, 136(1), 225–245. https://doi.org/10.1007/s11205-016-1528-6 29497233 10.1007/s11205-016-1528-6 PMC5816115

[bibr3-08862605211006352] Álvarez-García D., García T., & Núñez J. C. (2015). Predictors of school bullying perpetration in adolescence: A systematic review. *Aggression and Violent Behavior*, 23, 126–136. https://doi.org/10.1016/j.avb.2015.05.007

[bibr4-08862605211006352] Alves E., Correia S., Barros H., & Azevedo A. (2012). Prevalence of self-reported cardiovascular risk factors in Portuguese women: A survey after delivery. *International Journal of Public Health*, 57(5), 837–847. https://pubmed.ncbi.nlm.nih.gov/22314542/22314542 10.1007/s00038-012-0340-6

[bibr5-08862605211006352] Björkqvist K., Lagerspetz K. M., & Kaukiainen A. (1992). Do girls manipulate and boys fight? Developmental trends in regard to direct and indirect aggression. *Aggressive Behavior*, 18(2), 117–127. https://psycnet.apa.org/record/1992-30761-001

[bibr6-08862605211006352] Bowes L., Arseneault L., Maughan B., Taylor A., Caspi A., & Moffitt T. E. (2009). School, neighborhood, and family factors are associated with children’s bullying involvement: A nationally representative longitudinal study. *Journal of the American Academy of Child and Adolescent Psychiatry*, 48(5), 545–553. https://doi.org/10.1097/CHI.0b013e31819cb017. 19325496 10.1097/CHI.0b013e31819cb017 PMC4231780

[bibr7-08862605211006352] Christie-Mizell C. A. (2003). Bullying: The consequences of interparental discord and child’s self-concept. *Family Process*, 42(2), 237–251. https://onlinelibrary.wiley.com/doi/abs/10.1111/j.1545-5300.2003.42204.x 12879596 10.1111/j.1545-5300.2003.42204.x

[bibr8-08862605211006352] Coloroso B. (2008). *The bully, the bullied, and the bystander*. HarperCollins Publishers.

[bibr9-08862605211006352] Committee on the Biological and Psychosocial Effects of Peer Victimization. (2016). *Preventing bullying through science, policy, and practice*. National Academies Press. 27748087

[bibr10-08862605211006352] Craig W., & Harel-Fisch Y. (2004). *Bullying, physical fighting and victimization*.

[bibr11-08862605211006352] Craig W., Harel-Fisch Y., Fogel-Grinvald H., Dostaler S., Hetland J., Simons-Morton B., Molcho M., de Mato M. G., Overpeck M., Due P., Pickett W., Violence HBSC, Group Injuries Prevention Focus, & Bullying Writing Group. HBSC (2009). A cross-national profile of bullying and victimization among adolescents in 40 countries. *International Journal of Public Health*, 54(2), 216–224. https://doi.org/10.1007/s00038-009-5413-9 19623475 10.1007/s00038-009-5413-9 PMC2747624

[bibr12-08862605211006352] Davidson L. M., & Demaray M. K. (2007). Social support as a moderator between victimization and internalizing-externalizing distress from bullying. *School Psychology Review*, 36(3), 383–405. https://www.semanticscholar.org/paper/Social-Support-as-a-Moderator-Between-Victimization-Davidson-Demaray/45dd27874a421534d8b2523535c97ce03570fedc

[bibr13-08862605211006352] Eşkisu M. (2014). The relationship between bullying, family functions, perceived social support among high school students. *Procedia: Social and Behavioral Sciences*, 159, 492–496. https://doi.org/10.1016/j.sbspro.2014.12.412

[bibr14-08862605211006352] Espelage D. L., Holt M. K., & Henkel R. R. (2003). Examination of peer-group contextual effects on aggression during early adolescence. *Child Development*, 74(1), 205–220. https://doi.org/10.1111/1467-8624.00531 12625446 10.1111/1467-8624.00531

[bibr15-08862605211006352] Espelage D. L., & Swearer S. M. (2003). Research on school bullying and victimization: What have we learned and where do we go from here? *School Psychology Review*, 32(3), 365–383. https://doi.org/10.1080/02796015.2003.12086206

[bibr16-08862605211006352] Farrington D. P. (1993). *Understanding and preventing bullying*. University of Chicago Press.

[bibr17-08862605211006352] Felitti V. J., Anda R. F., Nordenberg D., Williamson D. F., Spitz A. M., Edwards V., Koss M. P., & Marks J. S. (1998). Relationship of childhood abuse and household dysfunction to many of the leading causes of death in adults: The adverse childhood experiences (ACE) study. *American Journal of Preventive Medicine*, 14(4), 245–258. https://pubmed.ncbi.nlm.nih.gov/9635069/9635069 10.1016/s0749-3797(98)00017-8

[bibr18-08862605211006352] Finkelhor D., Shattuck A., Turner H., & Hamby S. (2015). A revised inventory of adverse childhood experiences. *Child Abuse & Neglect*, 48, 13–21. https://doi.org/10.1016/j.chiabu.2015.07.011 26259971 10.1016/j.chiabu.2015.07.011

[bibr19-08862605211006352] Foster H., & Brooks-Gunn J. (2013). Neighborhood, family and individual influences on school physical victimization. *Journal of Youth and Adolescence*, 42(10), 1596–1610. https://doi.org/10.1007/s10964-012-9890-4 23263822 10.1007/s10964-012-9890-4 PMC3732577

[bibr20-08862605211006352] Frączek A., & Kirwil L. (1992). Family Life and Child Aggression: Studies on Some Socialization Conditions for Development of Aggression. In Frączek A., & Zumkley H. (Eds), *Socialization and Aggression. Recent Research in Psychology*. Springer. https://doi.org/10.1007/978-3-642-84653-3_10

[bibr21-08862605211006352] Garmy P., Vilhjálmsson R., & Kristjánsdóttir G. (2018). Bullying in school-aged children in Iceland: A cross-sectional study. *Journal of Pediatric Nursing*, 38, e30-e34. https://doi.org/10.1016/j.pedn.2017.05.009 10.1016/j.pedn.2017.05.009 28583432

[bibr22-08862605211006352] Glew G. M., Fan M. Y., Katon W., Rivara F. P., & Kernic M. A. (2005). Bullying, psychosocial adjustment, and academic performance in elementary school. *Archives of Pediatrics & Adolescent Medicine*, 159(11), 1026–1031. https://jamanetwork.com/journals/jamapediatrics/fullarticle/486162 16275791 10.1001/archpedi.159.11.1026

[bibr23-08862605211006352] Halfon N., Larson K., Son J., Lu M., & Bethell C. (2017). Income inequality and the differential effect of adverse childhood experiences in US children. *Academic Pediatrics*, 17(7s), S70– S78. https://doi.org/10.1016/j.acap.2016.11.007 28865663 10.1016/j.acap.2016.11.007

[bibr24-08862605211006352] Hamburger M. E., Basile K. C., & Vivolo A. M. (2011). *Measuring bullying victimization, perpetration, and bystander experiences; a compendium of assessment tools*. Createspace Independent Pub.

[bibr25-08862605211006352] Helsen M., Wilma V., & Wim M. (2000). Social support from parents and friends and emotional problems in adolescence. *Journal of Youth and Adolescence*, 29, 319–335. https://doi.org/10.1023/A:1005147708827

[bibr26-08862605211006352] Henderson S. E., Dowda R., & Robles-Piña R. A. (2018). Predictors of bullying behavior: An adlerian approach. *Bullying prevention and intervention at school* (pp. 17–35). Springer.

[bibr27-08862605211006352] Hertz M. F., Jones S. E., Barrios L., David-Ferdon C., & Holt M. (2015). Association between bullying victimization and health risk behaviors among high school students in the United States. *The Journal of School Health*, 85(12), 833–842. https://doi.org/10.1111/josh.12339 26522172 10.1111/josh.12339 PMC4721503

[bibr28-08862605211006352] Holt M. K., & Espelage D. L. (2007). Perceived social support among bullies, victims, and bully-victims. *Journal of Youth and Adolescence*, 36(8), 984–994. https://doi.org/10.1007/s10964-006-9153-3

[bibr29-08862605211006352] Hong J. S., & Espelage D. L. (2012). A review of research on bullying and peer victimization in school: An ecological system analysis. *Aggression and Violent Behavior*, 17(4), 311–322. https://doi.org/10.1016/j.avb.2012.03.003

[bibr30-08862605211006352] Instituto Nacional de Estatística. (2007). *População Estrangeira em Portugal–2006*. Instituto Nacional de Estatística.

[bibr31-08862605211006352] Jansen D., Veenstra R., Ormel J., Verhulst F., & Reijneveld S. (2011). Early risk factors for being a bully, victim, or bully/victim in late elementary and early secondary education. The longitudinal TRAILS study. *BMC Public Health*, 11(440), 1471–2458. https://doi.org/10.1186/1471-2458-11-440 10.1186/1471-2458-11-440 PMC3128024 21645403

[bibr32-08862605211006352] Jansen P., Verlinden M., Dommisse-van Berkel A., Mieloo C., van der Ende J., Veenstra R., Verhulst F. C., Jansen W., & Tiemeier H. (2012). Prevalence of bullying and victimization among children in early elementary school: Do family and school neighbourhood socioeconomic status matter? *BMC Public Health*, 12(494), 1471–2458. https://doi.org/10.1186/1471-2458-12-494 10.1186/1471-2458-12-494 PMC3575320 22747880

[bibr33-08862605211006352] Kitzmann K. M., Gaylord N. K., Holt A. R., & Kenny E. D. (2003). Child witnesses to domestic violence: A meta-analytic review. *Journal of Consulting and Clinical Psychology*, 71(2), 339–352. https://doi.org/10.1037/0022-006x.71.2.339 12699028 10.1037/0022-006x.71.2.339

[bibr34-08862605211006352] Klomek A. B., Sourander A., Niemelä S., Kumpulainen K., Piha J., Tamminen T., Almqvist F., & Gould M. S. (2009). Childhood bullying behaviors as a risk for suicide attempts and completed suicides: A population-based birth cohort study. *Journal of the American Academy of Child and Adolescent Psychiatry*, 48(3), 254–261. https://doi.org/10.1097/CHI.0b013e318196b91f 19169159 10.1097/CHI.0b013e318196b91f

[bibr35-08862605211006352] Kolk B. A. van der. (2017). Developmental Trauma Disorder: Toward a rational diagnosis for children with complex trauma histories. *Psychiatric Annals*, 35(5), 401–408. https://doi.org/10.3928/00485713-20050501-06

[bibr36-08862605211006352] Larsen P. S., Kamper-Jørgensen M., Adamson A., Barros H., Bonde J. P., Brescianini S., Eggesbø M., Brophy S., Casas M., Charles M. A., Devereux G., Fantini M. P., Frey U., Gehring U., Grazuleviciene R., Henriksen T. B., Hertz-Picciotto I., Hertz-Picciotto B., Hryhorczuk D. O., & Nybo Andersen A. M. (2013). Pregnancy and birth cohort resources in Europe: A large opportunity for aetiological child health research. *Paediatric and Perinatal Epidemiology*, 27(4), 393–414. https://doi.org/10.1111/ppe.12060 23772942 10.1111/ppe.12060

[bibr37-08862605211006352] Lereya S. T., Copeland W. E., Costello E. J., & Wolke D. (2015). Adult mental health consequences of peer bullying and maltreatment in childhood: Two cohorts in two countries. *The Lancet Psychiatry*, 2(6), 524–531. https://www.thelancet.com/journals/lanpsy/article/PIIS2215-0366(15)00165-0/fulltext 26360448 10.1016/S2215-0366(15)00165-0 PMC4580734

[bibr38-08862605211006352] Luthar S. S., Cicchetti D., & Becker B. (2000). The construct of resilience: A critical evaluation and guidelines for future work. *Child Development*, 71(3), 543–562. https://doi.org/10.1111/1467-8624.00164 10953923 10.1111/1467-8624.00164 PMC1885202

[bibr39-08862605211006352] Magklara K., Skapinakis P., Gkatsa T., Bellos S., Araya R., Stylianidis S., & Mavreas V. (2012). Bullying behaviour in schools, socioeconomic position and psychiatric morbidity: A cross-sectional study in late adolescents in Greece. *Child and Adolescent Psychiatry and Mental Health*, 6, 8–8. https://www.ncbi.nlm.nih.gov/pmc/articles/PMC3298787/22325708 10.1186/1753-2000-6-8 PMC3298787

[bibr40-08862605211006352] Manning M., Heron J., & Marshall T. (1978). Styles of hostility and social interactions at nursery, at school, and at home. an extended study of children. *Book Suppl J Child Psychol Psychiatr*, 1, 29-58. 670342

[bibr41-08862605211006352] Mazur J., Tabak I., & Zawadzka D. (2017). Determinants of bullying at school depending on the type of community: Ecological analysis of secondary schools in Poland. *School Mental Health*, 9(2), 132–142. https://doi.org/10.1007/s12310-017-9206-7 28572858 10.1007/s12310-017-9206-7 PMC5429380

[bibr42-08862605211006352] Moore J. C., Stinson L. L., & Welniak E. J. (2000). Income measurement error in surveys: A review. *Journal of Official Statistics*, 16(34), 331–361.

[bibr43-08862605211006352] Moore S. E., Norman R. E., Suetani S., Thomas H. J., Sly P. D., & Scott J. G. (2017). Consequences of bullying victimization in childhood and adolescence: A systematic review and meta-analysis. *World Journal of Psychiatry*, 7(1), 60. https://doi.org/10.5498/wjp.v7.i1.60 10.5498/wjp.v7.i1.60 PMC5371173 28401049

[bibr44-08862605211006352] Nansel T. R., Craig W., Overpeck M. D., Saluja G., & Ruan W. J. (2004). Cross-national consistency in the relationship between bullying behaviors and psychosocial adjustment. *Archives of Pediatrics & Adolescent Medicine*, 158(8), 730–736. https://doi.org/10.1001/archpedi.158.8.730 15289243 10.1001/archpedi.158.8.730 PMC2556236

[bibr45-08862605211006352] Nansel T. R., Overpeck M., Pilla R. S., Ruan W. J., Simons-Morton B., & Scheidt P. (2001). Bullying behaviors among US youth: Prevalence and association with psychosocial adjustment. *JAMA*, 285(16), 2094–2100. https://www.ncbi.nlm.nih.gov/pmc/articles/PMC2435211/11311098 10.1001/jama.285.16.2094 PMC2435211

[bibr46-08862605211006352] Nordhagen R., Nielsen A., Stigum H., & Kohler L. (2005). Parental reported bullying among Nordic children: A population-based study. *Child Care Health and Development*, 31(6), 693–701. https://doi.org/10.1111/j.1365-2214.2005.00559.x 16207227 10.1111/j.1365-2214.2005.00559.x

[bibr47-08862605211006352] Olweus D. (1993). Bully/victim problems among schoolchildren: Long-term consequences and an effective intervention program. In *Mental disorder and crime* (pp. 317–349). SAGE Publications.

[bibr48-08862605211006352] Ondersma S. J. (2002). Predictors of neglect within low-SES families: The importance of substance abuse. *American Journal of Orthopsychiatry*, 72(3), 383–391. https://doi.org/10.1037/0002-9432.72.3.383 15792050 10.1037/0002-9432.72.3.383

[bibr49-08862605211006352] Rothon C., Head J., Klineberg E., & Stansfeld S. (2011). Can social support protect bullied adolescents from adverse outcomes? A prospective study on the effects of bullying on the educational achievement and mental health of adolescents at secondary schools in East London. *Journal of Adolescence*, 34(3), 579–588. https://doi.org/10.1016/j.adolescence.2010.02.007 20637501 10.1016/j.adolescence.2010.02.007 PMC3107432

[bibr50-08862605211006352] Schaffer D. (1994). *Social and personality development*. Wadworth.

[bibr51-08862605211006352] Shields A., & Cicchetti D. (2001). Parental maltreatment and emotion dysregulation as risk factors for bullying and victimization in middle childhood. *Journal of Clinical Child & Adolescent Psychology*, 30(3), 349–363. https://pubmed.ncbi.nlm.nih.gov/11501252/10.1207/S15374424JCCP3003_7 11501252

[bibr52-08862605211006352] Silva-Rocha N., Soares S., Brochado S., & Fraga S. (2020). Bullying involvement, family background, school life, and well-being feelings among adolescents. *Journal of Public Health*, 28(5), 481–489. https://doi.org/10.1007/s10389-019-01076-2

[bibr53-08862605211006352] Sourander A., Brunstein Klomek A., Kumpulainen K., Puustjärvi A., Elonheimo H., Ristkari T., Moilanen I., Tamminen T., Piha J., & Ronning J. A. (2011). Bullying at age eight and criminality in adulthood: Findings from the Finnish Nationwide 1981 Birth Cohort Study. *Social Psychiatry and Psychiatric Epidemiology*, 46(12), 1211–1219. https://doi.org/10.1007/s00127-010-0292-1 21120451 10.1007/s00127-010-0292-1

[bibr54-08862605211006352] Sourander A., Jensen P., Rönning J. A., Niemelä S., Helenius H., Sillanmäki L., Kumpulainen K., Piha J., Tamminen T., Moilanen I., & Almqvist F. (2007). What is the early adulthood outcome of boys who bully or are bullied in childhood? The Finnish “From a Boy to a Man” study. *Pediatrics*, 120(2), 397–404. https://doi.org/10.1542/peds.2006-2704 17671067 10.1542/peds.2006-2704

[bibr55-08862605211006352] Sourander A., Ronning J., Brunstein-Klomek A., Gyllenberg D., Kumpulainen K., Niemelä S., Helenius H., Sillanmäki L., Piha J., Tamminen T., Moilanen I., Almqvist F., Ristkari T., & Almqvist F. (2009). Childhood bullying behavior and later psychiatric hospital and psychopharmacologic treatment: Findings from the Finnish 1981 birth cohort study. *Archives of General Psychiatry*, 66(9), 1005–1012. https://doi.org/10.1001/archgenpsychiatry.2009.122 19736357 10.1001/archgenpsychiatry.2009.122

[bibr56-08862605211006352] Steinfeldt J. A., Vaughan E. L., LaFollette J. R., & Steinfeldt M. C. (2012). Bullying among adolescent football players: Role of masculinity and moral atmosphere. *Psychology of Men & Masculinity*, 13(4), 340–353. https://psycnet.apa.org/record/2012-01737-001

[bibr57-08862605211006352] Stevens V., Bourdeaudhuij I., & Ost P. (2002). Relationship of the family environment to children’s involvements in bully/ victim problems at school. *Journal of Youth and Adolescence Volume*, 31(6), 419–428. https://link.springer.com/article/10.1023/A:1020207003027

[bibr58-08862605211006352] Tippett N., & Wolke D. (2014). Socioeconomic status and bullying: A meta-analysis. *American Journal of Public Health*, 104(6), e48–e59. https://www.ncbi.nlm.nih.gov/pmc/articles/PMC4061998/10.2105/AJPH.2014.301960 PMC4061998 24825231

[bibr59-08862605211006352] Ttofi M., & Farrington D. (2009). What works in preventing bullying: Effective elements of anti-bullying programmes. *Journal of Aggression, Conflict and Peace Research*, 1(1), 13–24. https://doi.org/10.1108/17596599200900003

[bibr60-08862605211006352] Ttofi M. M., Farrington D. P., Lösel F., & Loeber R. (2011). The predictive efficiency of school bullying versus later offending: A systematic/meta-analytic review of longitudinal studies. *Criminal Behaviour and Mental Health*, 21(2), 80–89. https://doi.org/10.1002/cbm.808 21370293 10.1002/cbm.808

[bibr61-08862605211006352] Unesco Institute for Statistics. (2012). *International standard classification of education - ISCED 2011*. http://uis.unesco.org/sites/default/files/documents/international-standard-classification-of-education-isced-2011-en.pdf

[bibr62-08862605211006352] UNICEF. (2018). *An everyday lesson: #ENDviolence in schools*. UNICEF. https://www.unicef.org/media/73516/file/An-Everyday-Lesson-ENDviolence-in-Schools-2018.pdf.pdf

[bibr63-08862605211006352] Waasdorp T. E., Mehari K. R., Milam A. J., & Bradshaw C. P. (2018). Health-related risks for involvement in bullying among middle and high school youth. *Journal of Child and Family Studies*, 28, 2606–2617. https://link.springer.com/article/10.1007/s10826-018-1260-8

[bibr64-08862605211006352] Walsh D., McCartney G., Smith M., & Armour G. (2019). Relationship between childhood socioeconomic position and adverse childhood experiences (ACEs): A systematic review. *Journal of Epidemiology and Community Health*, 73(12), 1087–1093. https://doi.org/10.1136/jech-2019-212738 31563897 10.1136/jech-2019-212738 PMC6872440

[bibr65-08862605211006352] Wang J., Iannotti R. J., & Luk J. W. (2011). Peer victimization and academic adjustment among early adolescents: Moderation by gender and mediation by perceived classmate support. *Journal of School Health*, 81(7), 386–392. https://doi.org/10.1111/j.1746-1561.2011.00606.x 21668878 10.1111/j.1746-1561.2011.00606.x

[bibr66-08862605211006352] World Health Organization. (2017). *Child and adolescent health*. World Health Organization.

[bibr67-08862605211006352] Yeager D. S., Fong C. J., Lee H. Y., & Espelage D. L. (2015). Declines in efficacy of anti-bullying programs among older adolescents: Theory and a three-level meta-analysis. *Journal of Applied Developmental Psychology*, 37, 36–51. https://doi.org/10.1016/j.appdev.2014.11.005

[bibr68-08862605211006352] Zarling A. L., Taber-Thomas S., Murray A., Knuston J. F., Lawrence E., Valles N. L., DeGarmo D. S., & Bank L. (2013). Internalizing and externalizing symptoms in young children exposed to intimate partner violence: Examining intervening processes. *Journal of Family Psychology*, 27(6), 945–955. https://pubmed.ncbi.nlm.nih.gov/24294933/24294933 10.1037/a0034804 PMC5308783

